# The Effects of Essential and Non-Essential Metal Toxicity in the *Drosophila melanogaster* Insect Model: A Review

**DOI:** 10.3390/toxics9100269

**Published:** 2021-10-16

**Authors:** Mitchell R. Slobodian, Jesse D. Petahtegoose, Athena L. Wallis, Danica C. Levesque, Thomas J. S. Merritt

**Affiliations:** Faculty of Science and Engineering, Laurentian University, 935 Ramsey Lake Rd, Sudbury, ON P3E 2C6, Canada; mslobodian@laurentian.ca (M.R.S.); jd_petahtegoose@laurentian.ca (J.D.P.); twallis@laurentian.ca (A.L.W.); dlevesque3@laurentian.ca (D.C.L.)

**Keywords:** essential metals, non-essential metals, *Drosophila melanogaster*, metal toxicity, iron homeostasis, zinc homeostasis, copper homeostasis, metallothioneins, metal transcription factor

## Abstract

The biological effects of environmental metal contamination are important issues in an industrialized, resource-dependent world. Different metals have different roles in biology and can be classified as essential if they are required by a living organism (e.g., as cofactors), or as non-essential metals if they are not. While essential metal ions have been well studied in many eukaryotic species, less is known about the effects of non-essential metals, even though essential and non-essential metals are often chemically similar and can bind to the same biological ligands. Insects are often exposed to a variety of contaminated environments and associated essential and non-essential metal toxicity, but many questions regarding their response to toxicity remain unanswered. *Drosophila melanogaster* is an excellent insect model species in which to study the effects of toxic metal due to the extensive experimental and genetic resources available for this species. Here, we review the current understanding of the impact of a suite of essential and non-essential metals (Cu, Fe, Zn, Hg, Pb, Cd, and Ni) on the *D. melanogaster* metal response system, highlighting the knowledge gaps between essential and non-essential metals in *D. melanogaster*. This review emphasizes the need to use multiple metals, multiple genetic backgrounds, and both sexes in future studies to help guide future research towards better understanding the effects of metal contamination in general.

## 1. Introduction

Metal ions present an interesting challenge of balance in biology: many are indispensable, but all can be very toxic. Organisms have evolved a diverse suite of mechanisms to accomplish this balancing act. With respect to their effects in biological systems, metals can be separated into two categories: essential and non-essential. Essential metal ions function as protein cofactors in a wide variety of biological processes, are non-toxic when present in trace amounts in an organism, but can be toxic if concentrations exceed a threshold [1]. Non-essential metals have no biological function and are toxic to the organism in even trace amounts. Whether a metal is essential or non-essential, exposure to elevated amounts of either can cause serious adverse health effects. Toxic environmental exposure is especially of concern in mining-, or other industrial resource-based, communities around the world, where elevated levels of toxic metals, such as copper (Cu) and nickel (Ni), are detectable even far from the original source of contamination [2,3].Given these concerns, the toxic effects on biological systems of metals, and especially non-essential metals, are surprisingly under-studied, especially the combined effects of multiple metals and the variability in response between the sexes and with genetic variation.

The toxic effects of essential and non-essential metals are complex and varied with a suite of molecular mechanisms including the production of Reactive Oxygen Species (ROS), DNA binding and cleavage, and replacement of essential metal ions with non-essential metal ions in metalloproteins [4]. Metal exposure leads to a variety of different responses reflecting the specific chemical properties of a metal ion [4,5]. Further, different populations of organisms often respond differently to metal toxicity due to adaptations to their local environment. For example, populations sampled near a nickel mine may show fewer toxic effects following Ni exposure than populations sampled from relatively clean environments [6,7,8,9,10,11,12].

In this review, we focus on the model organism *D. melanogaster*, the common fruit fly, as a model for toxic metal response. *D. melanogaster* is as an excellent model organism due to its fully sequenced genome, a wealth of genetic and genomic experimental and analytical tools, and ease of use in laboratory studies [13]. Metal toxicity can be induced simply by adding metals to fly food. Populations of *D. melanogaster* can be found across the globe allowing investigation of local adaptations and diversity [14,15,16,17,18,19]. Further, the small size and easy care of *D. melanogaster* translates into large sample sizes that include males, females, and a diversity of genetic backgrounds.

Overall, the biology of essential metals is better understood than that of non-essential metals. The essential metals Cu, iron (Fe), and zinc (Zn) are the most abundant trace elements present in animals and their systems are well studied in many organisms. In contrast, non-essential metals are much less well studied, although they are often central players in environmental contamination [20]. Non-essential metal ions are chemically similar to their essential metal counterparts and can have similar interactions with the proteins and pathways that regulate essential metal ions, although generally disrupting, rather than enabling, these systems [21,22]. For example, dyshomeostasis (systemic imbalance) in Fe, Zn, and Cu impacts not only each of their respective regulatory pathways, but proteins in other essential metal pathways [20,23].

Here, we review the biology of metal toxicity in *D. melanogaster*, highlighting the current knowledge in both essential and non-essential metal ion homeostasis and to identifying gaps in knowledge that require future research.

## 2. Essential and Non-Essential Metals

Environmentally relevant non-essential metal ions can interact with essential metal proteins and pathways, reflecting the many chemical and toxicological similarities between essential and non-essential metals. Both essential and non-essential metals produce toxic effects in *D. melanogaster* by creating ROS, directly or indirectly, as well as a myriad of genotoxic effects such as cleaving DNA [4,24]. Many essential and non-essential metals are borderline hard/soft metals and are, therefore, capable of binding to hard bases such as oxygen and peroxide, or soft bases such as sulfur, leading to oxidative stress damage [25,26]. The same metals are borderline Lewis acids [27] and can bind to histidine residues and nitrogen, leading to damage through protein misfolding [28]. In addition, some essential and non-essential metal ions have similar chemical properties [21,22]. The combination of these three characteristics allow many metals to interact with a huge variety of biological ligands and be biologically active even if the metal itself is not required by the organism [21,22,27,28]. For example, Cu is an essential element in *D. melanogaster* while Ni is non-essential, but both ions have a 2+ charge and are adjacent on the periodic table with similar atomic properties. This similarity is reflected in their binding capabilities: both have similar metal ligand bond strength, and both often have similar final structures after interaction with the same ligand [22]. As another example, Cu is the natural cofactor for the *D. melanogaster* ROS scavenger enzyme superoxide dismutase 1 (SOD1), but Ni can bind to the enzyme, replacing Cu and inhibiting enzyme activity [29]. Both Cu and Ni produce ROS and both metals cause indirect DNA damage at toxic concentrations, but only Cu, and not Ni, interacts with SOD1 to produce a functional enzyme. These examples reflect the chemical similarly and interwoven biology of essential and non-essential metals and underscore the need to study both.

## 3. Metal Homeostasis in *Drosophila melanogaster*

**Figure 1:**
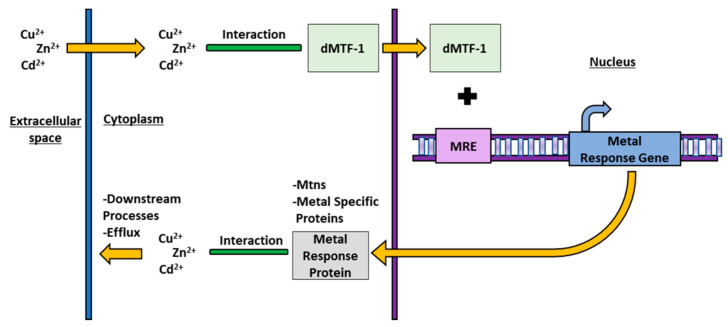
A diagram of the *D. melanogaster* general metal response system responds to both essential and non-essential metal stress. All figures follow the same pattern and colour scheme: the movement of metal ions is represented by orange arrows, interaction between metals and proteins is represented by green bars/arrows, transcription factor proteins are coloured light green, response element proteins are light purple, DNA is purple, genes are dark blue, and other proteins are gray. Underlined labels denote areas inside the cell, organelles, or space outside of the cell.

Metal response in *D. melanogaster* is mediated by a diverse suite of proteins, each with varying degrees of specificity. Fundamentally, there are two different types of metal response pathways; a general metal response pathway, which responds to toxic conditions for both essential and non-essential metals, and an essential metal specific response pathways to transport and regulate homeostasis of a particular essential metal ion [30,31]. The first step in the general metal response pathway is activated when a metal enters the cytoplasm and binds to Metal Transcription Factor 1 (MTF-1) [32]. In the second step, the MTF-1 protein is then localized to the nucleus of the cell where it binds to a Metal Response Element (MRE) consensus sequence [30,31,33]. In the third, and final, step in this pathway this binding activates transcription of a downstream metallothionein (Mtn) gene or other metal response gene [34,35]. Metallothioneins are small non-specific metal binding proteins which bind both essential and non-essential metal ions and transport them out of the cell for detoxification [36]. The *D. melanogaster* general metal response pathway is shown below in Figure 1. Although the specific mechanisms governing the general metal response pathway are still unknown, the signaling cascade of MTF-1 binding to *MRE* leading to transcription of *Mtn* genes is present in all eukaryotes [37].

The second type of metal response pathways is specific to a particular essential metal ion, such as Cu, Zn, and Fe, and includes specific importers, exporters, and chaperone proteins that regulate homeostasis [38]. Many of the proteins in each of these pathways are specific to only the one essential metal, but as we discuss later in the review some proteins interact with multiple, essential and non-essential, metals. Both the general metal response proteins and essential metal specific pathways are evolutionarily conserved, and many *D. melanogaster* proteins have homologs in other eukaryotes, including humans [39,40,41]. The broad evolutionary conservation of metal response pathways makes *D. melanogaster* an effective model for exploring the impact of essential and non-essential toxicity on metal homeostasis.

## 4. MTF-1 in *Drosophila melanogaster*

### 4.1. dMTF-1 Activation and Mechanism

The transcription factor *Drosophila* Metal Transcription Factor (dMTF-1) is required to regulate stress responses from both essential and non-essential metals. The first step in the *D. melanogaster* metal response cascade occurs when dMTF-1 activates in response to changing metal ion concentrations in the cytosol of the cell [32]. The structure of dMTF-1 is key to the interaction of the protein with both essential and non-essential metals. dMTF-1 is a single 791 amino acid chain with a molecular mass of 85 kDa [42] that includes an active site of six Cys_2_-His_2_ zinc finger regions [39,42]. These zinc finger regions interact with unbound metal ions in the cell cytosol to signal dMTF-1 to translocate to the nucleus and bind to *MRE* [43]. The mechanism by which free metal ions interact with the dMTF-1 zinc finger regions is currently not understood [32,37,44], but two models have been proposed, both focused on the histidine residues in the zinc finger regions. Translocation may occur following phosphorylation or dephosphorylation of dMTF-1 [37,45], or interactions between free metal ions and Zn molecules in the zinc finger region [46,47]. Histidine residues interact with borderline Lewis acids, including Cu and Ni, in other proteins [22] suggesting similar interactions here. In addition to interactions with metal ions, dMTF-1 accumulates in the nucleus following oxidative stress, again through a poorly understood mechanism, possibly involving indirect effects driven by the ROS generated by the metal ions [36,48,49]. Regardless of the activation mechanism, however, nuclear dMTF-1 can bind to an *MRE* [42,50,51]. *MREs* are located upstream of genes that code for proteins which control metal ion homeostasis in many organisms, including *D. melanogaster* [34,35]. The large diversity of metal response proteins allows the cell to produce the appropriate metallothionein or metal-specific protein to effectively regulate metal homeostasis [52,53]. The genes downstream of *MRE*s are tightly regulated, as over or under expression of downstream metal response genes can lead to sensitivity to toxic metals or genotoxicity [53,54]. Through this binding, dMTF-1 activates the transcription for a vast suite of proteins with a large range of functions and selectivity and a more complete understanding of its mechanisms is vital for a better understanding of essential and non-essential metal regulation.

### 4.2. dMTF-1 and Homeostasis during Metal Stress

dMTF-1 responds to both accumulation and depletion of essential metal ions and is required to regulate stress caused by metal exposure. Knockout for the *dMTF-1* gene significantly shortened lifespan in flies exposed to small amounts of either Cu or Zn [42,50,51]. Flies with a knockout mutation in dMTF-1 do not transcribe Mtn genes and the shortened lifespan with Cu exposure is likely a function of the toxic unbound metal ions. Interestingly, dMTF-1 is also necessary for regulating essential metal homeostasis in situations where stress is caused by a lack of essential metals. For example, the combination of knocking out *dMTF-1* and addition of a Cu chelator to *D. melanogaster* food, and the resulting reduction in available Cu, resulted in a significant decrease in survival [50]. The mechanism through which dMTF-1 differentiates between binding to an MRE upstream of an Mtn during various toxic metal conditions, and binding to a different MRE upstream of an importer during starvation is likely through differences in phosphorylation of dMTF-1 [33,34], and metal-specific binding to dMTF-1 [55]. dMTF-1 is differentially phosphorylated in periods of metal stress and metal starvation, leading to binding of different MREs [56]. The complex role of this protein in maintaining homeostasis highlights the need for research including periods of both excess and starvation.

### 4.3. dMTF-1 and Essential Metals

dMTF-1 is an important signalling protein and transcription factor in the homeostasis of many essential metals including Cu, Zn, and Fe. dMTF-1 helps to maintain essential metal homeostasis by binding to many genes in each of the essential metal pathways. DmATP7 is a Cu transport protein which is necessary for uptake and efflux of Cu in *D. melanogaster* [57] and is essential for larval development. *DmATP7* expression is upregulated in *D. melanogaster* when exposed to Cu load, but only in the presence of a functional dMTF-1. When flies are knocked out for *dMTF-1*, background expression of *DmATP7* is maintained in the cells, but upregulation of *DmATP7* is absent in the knockout flies following exposure to Cu [58]. This complex interaction suggests that *DmATP7* expression is independent of *dMTF-1,* and dMTF-1′s role in this interaction is to primarily maintain essential metal homeostasis in toxic conditions [58]. dMTF-1 also regulates the transcription of Cu importer protein Ctr1B in situations of Cu specific stress [50,59,60]. The dMTF-1 protein is activated by exposure to Cu depleted food and upregulates transcription of *Ctr1B* to create more Ctr1B importers [50,51,59]. dMTF-1 knockout flies do not upregulate *Ctr1B* during Cu depletion, eliminating their ability to increase Cu uptake in the cell, and leading to a significant decrease in survival and a significant increase in development time [59,61].

The essential metal Zn, similar to Cu, is tightly regulated by dMTF-1 signaling in *D. melanogaster* [62]. Members of the ZnT protein family (efflux) and the Zip protein family (intake) help control the uptake and efflux of Zn into the cytosol [63]. However, only the Zn exporters are regulated by the concentration gradient of dMTF-1 between the cytosol and nucleus [35]. Zn toxicity results in upregulation of *dMTF-1* expression and translocation of dMTF-1 into nucleus where the protein binds to the *MRE* upstream of *ZnT* increasing transcription of *ZnT* and production of more Zn exporter protein [48]. Currently no Zn importers have been identified as targets of dMTF-1 in *D. melanogaster* but, given that regulation of other metal importers are regulated by dMTF-1, it is possible that some Zn importers may be regulated by it as well. The mechanism of regulation of Zn exporters in periods of Zn excess and depletion allows dMTF-1 to maintain metal homeostasis.

Interestingly, the importance of dMTF-1 in essential metal regulation differs between metals. For example, Fe is another essential metal for *D. melanogaster*, but unbound Fe ions are extremely toxic due to their redox potential and Fe metabolism is tightly regulated. *Ferritin* codes for an Fe storage protein and is induced by dMTF-1 in the presence of Cu or Zn toxicity, but not Fe toxicity, suggesting that Fe regulation is independent of dMTF-1 [35]. Ferritin may play a role in general metal detoxification, however, potentially by releasing Fe to compete with unbound Cu and Zn ions or by using ferritin as temporary storage for Cu and Zn ions before being removed from the cell [35]. dMTF-1 has a central role in maintaining the balance for many essential metals and in the regulation of a broad suite of genes that facilitate metal homeostasis. More research is needed to determine the mechanism of activation for dMTF-1 in situations of essential metal stress and depletion.

### 4.4. dMTF-1 and Non-Essential Metals

dMTF-1 has a central role in maintaining homeostasis of non-essential metals, as well as essential metals. Non-essential metals are primarily regulated and eliminated by metallothioneins, but other metal binding proteins are sometimes involved [64]. Non-essential metals have no natural biological role and toxicity appears to be regulated in a simpler manner than essential metals, likely as the metals simply must be removed, not balanced. The non-essential metal cadmium (Cd) is often used as a toxicant in *D. melanogaster* essential metal toxicity studies; exposure to even small amounts of Cd leads to upregulation of *dMTF-1* expression [42,51]. Combining a *dMTF-1* mutation with Cd exposure significantly increases mortality [50,60]. While there are no Cd-specific proteins or transport systems activated by dMTF-1, Cd exposure does induce the production of other metal specific proteins, such as Ctr1B [64]. Interestingly, Ctr1B does not appear to interact directly with the Cd ions, but instead queues the import of other metal ions that balance out, or compete with, Cd ions [64]. While our understanding of Cd stress in *D. melanogaster* is incomplete, we know even less about the biology of other non-essential metals. Given the interaction with dMTF-1 and Cd, there is likely overlap in the biology of other understudied non-essential metals. For example, given their similar chemistry, Ni ions may interact with the histidine residues in the conserved zinc-finger region of dMTF-1 [22]. Future research including other non-essential metals could broaden our understanding of these environmentally relevant metals.

## 5. Metallothioneins in *Drosophila melanogaster*

### 5.1. Metallothionein Structure and Binding

The third step in the *D. melanogaster* metal response cascade is the production of metallothioneins [36]. Mtns are a family of small proteins, only 24–85 amino acids long, that are vital to chelate metal ions in situations of toxic metal stress [65]. Strikingly, cysteine residues make up 15–33% of the Mtn amino acid sequence and form the Mtn active site where they bind metal ions through metal thiolate bonds [65,66]. These bonds are less specific than the interactions in other metal-specific response proteins, allowing Mtns to bind to a broad suite of metals and restore homeostasis after a variety of stress responses [67,68]. The large number of cysteines provide multiple metal ion binding sites, allowing a single Mtn to transport multiple metal ions at once, dramatically increasing the efficiency of Mtn in chelating toxic metal ions [67,68]. Binding multiple metal ions allows for quick response to toxic conditions, which is especially important in situations of acute toxicity in which the increase in metal ions in the cell can be severe. The efficient and non-specific binding capabilities of Mtns is central to their role in detoxification of essential and non-essential metals.

### 5.2. Metallothionein Function

Metallothioneins have a central role in maintaining metal ion homeostasis *D. melanogaster* and mutations in the *D. melanogaster Mtn* genes result in significant decreases in survival under high-concentration Cu or Cd stress [52,54,69,70]. Interestingly, as we have seen in some of the other response systems, *Mtn* genes are upregulated in situations of essential metal starvation, as well as metal contamination [50,54], although it is unknown how activation of transcription occurs or what the biological impact of that upregulation is. Surprisingly, given this upregulation response, flies with knockout mutations in *Mtn* genes survive on essential metal starved medium [52], making the role of *Mtn* in response to essential metal starvation unclear. It is possible that starvation of one essential metal increases the concentration of another essential metal [71,72] which in turn induces the expression of *Mtn*, meaning that *Mtn* upregulation is the result of crosstalk. Overall, even with these uncertainties, it is clear that metallothioneins are important in maintaining essential and non-essential metal ion homeostasis.

### 5.3. Conservation of Mtn between Species

Metallothionein function is conserved across distantly related species, and the proteins have similar roles in metal detoxification species as distantly related as flies, fish, mice, and humans [41,73,74]. The conserved function is reflected in the conservation of the cysteine-rich active site across a broad suite of species [75]. Interestingly, even with the high degree of conservation, it is difficult to resolve the *Mtn* family evolutionary phylogeny, largely due to multiple *Mtn* isoforms [75,76]. These isoforms differ between species, with different species having different loci that are likely metal- and/or tissue-specific [77,78] and the result of gene or genome duplication events, although the timing of these events is unclear [75,76]. Determination of an accurate phylogeny is also complicated by the relatively large amounts of sequence variation outside of the active site and the small size of the proteins which limits the amount of information available for phylogenetic comparison [40]. Without an accurate gene phylogeny, it is difficult to distinguish orthologous genes from paralogous genes, limiting specific functional comparisons between species [41,74]. Nevertheless, the conservation of both the function of Mtn, and the active site of Mtn, across eukaryotes does allow for broad conclusions to be drawn regarding the roles of Mtn in the biology of essential and non-essential metals.

### 5.4. Drosophila melanogaster Mtn and Essential Metal Stress

In *D. melanogaster*, Mtns are produced in response to stressful conditions created by disruptions in essential metal homeostasis. Multiple Mtn isoforms respond in metal-specific fashion allowing *D. melanogaster* to respond to the wide array of metal contaminants [31]. There are currently six known *D. melanogaster Mtn* genes, *MtnA, B, C, D, E,* and *F*, each coding for a different Mtn isoform, MtnA–F [40,54,69] and the isoforms vary in metal and tissue specificity.

Isoform expression increases following essential metal stress, with the level of expression for each isoform varying depending on the tissue and metal [54,79]. For example, *MtnA* expression is significantly upregulated when *D. melanogaster* are exposed to Cu toxicity [54,70]. The other isoforms are upregulated in response to Cu toxicity as well, but none have as great of an increase in expression as *MtnA* [52,54]. Furthermore, knockout of *MtnA* significantly reduces survival of *D. melanogaster* on Cu supplemented food [52]. Double *MtnA* and *MtnB* knockout flies also have reduced survival when placed on Cu supplemented food; this reduction can be rescued by expression of either *MtnA* or *MtnB*, but, interestingly, *MtnA* has a much larger effect (60% rescue) than *MtnB* rescue (30%) [52]. This isoform specificity for Cu homeostasis suggests that other isoforms will have an affinity for other metals, but this has yet to be shown. *MtnB* is upregulated in response to other essential metals such as Zn [35], but the upregulation is less pronounced than for *MtnA* and Cu. *MtnB* expression is also induced by Fe, but its role in detoxification appears to be limited, and the Fe ions may induce *MtnB* expression indirectly by disrupting Zn homeostasis, rather than inducing expression on their own [80]. It is possible that *MtnB* is expressed preferentially as a response to non-essential metals instead, as it is required for survival when flies are exposed to Cd or mercury (Hg) [64].

In contrast to the largely distinct roles of *MtnA* and *MtnB*, *MtnC* and *MtnD* appear to have similar roles in metal response. Both genes are upregulated after flies are exposed to essential metals, although not to the same degree as *MtnA* or *MtnB* [52,54,78]. Further, overexpression of *MtnC* and *MtnD* is unable to rescue flies with *MtnA* or *MtnB* knockout mutations when placed on metal contaminated food [54] suggesting these genes have a smaller role in responding to essential metal toxicity. The role of *MtnE* is also unclear. Similar to *MtnC* and *MtnD*, *MtnE* is upregulated in response to essential metal stress, but less so than *MtnA* and *MtnB* [69,81] However, unlike *MtnC* and *MtnD*, *MtnE* is upregulated in flies knocked out for *MtnA–D* [69]**,** and these multiple knockout flies survive on metal supplemented food, suggesting that *MtnE* may act as a fail-safe responder [69,81].

The study of *D. melanogaster* metallothioneins is still new and expanding, *MtnE* was characterized in 2011, and the sixth isoform *MtnF* was discovered in 2020 [40]. Experimental data on *MtnF* is lacking, but sequence comparative analysis with the other *Mtn* genes predicts that *MtnF* could have a higher Zn specificity than the other isoforms [40]. Overall, while it is clear that the multiple *D. melanogaster* Mtn isoforms work together to detoxify essential metal stress, the precise role of each isoform remains unclear. It is also possible that more genes, either specific or general, have yet to be discovered, as two of the six currently known isoforms have been discovered in the past 10 years, and more research is needed to determine the full suite of *D. melanogaster* metallothioneins and their substrate specificities.

### 5.5. Sexual Dimorphism in Metallothionein Expression

The sex of an organism can have a major influence on its biology, including its metal response. For example, *Mtn* expression and Cu response is sex-specific in *D. melanogaster* [82]. Wildtype male flies have twice the level of *Mtn* expression than female flies [83], and male flies survive exposure to Cu toxicity significantly better than females, but this difference is dependent on genetic background. This complicated interaction between sex and genetic background highlights the importance of both in metal toxicity responses and biology in general [83] and the need for more research incorporating both.

### 5.6. Mtn and Non-Essential Stress

As we saw for dMTF-1, the understanding of *D. melanogaster Mtn* response to non-essential metals is much more limited than that of essential metals. Cd is the best studied of the group, followed by lead (Pb) and Hg. Similar to the essential metal responses, different Mtn proteins have different roles in non-essential metal response. The MtnB protein is particularly central in the non-essential metal response. *MtnB* mutations are lethal, or reduce survivability when combined with Cd or Pb toxicity, while mutations in the other *Mtn* genes have less pronounced effects [52,69,84], suggesting that, similar to the situation with the essential metals, *MtnC* and *MtnD* have smaller roles in the response to metal toxicity. Further, *MtnA* and *MtnB* are both upregulated by exposure to Hg toxicity [84]. *Mtn* expression is upregulated in response to Hg in mammals both directly and indirectly through other pathways depending on the tissue type [85], so it is possible that upregulation in *D. melanogaster* is also indirect. *Mtn* isoforms are also upregulated in response to non-essential metal stress in grasshoppers [86] showing conservation in function across species.

Research on non-essential metals and metallothioneins in *D. melanogaster* has predominantly been limited to Pb, Cd, and Hg. Many metals, including Ni, a major environmental contaminant, have essentially not been explored in flies. The Mtn genes in other organisms, from mammals to insects, do respond to Ni stress [87,88]. The broad conservation in response, and the high conservation of the active site and function of Mtn between *D. melanogaster* and other species, suggests that the *D. melanogaster* genes do respond to Ni stress as well. As with essential metals, future work investigating the effects of non-essential metals on *D. melanogaster* should be undertaken to determine if there is Mtn affinity to a metal ion, and to discover potential new *Mtn* isoforms which could have an impact on non-essential metal detoxification in *D. melanogaster*.

## 6. Metal Transport Systems in *Drosophila melanogaster*

In addition to the general metal response systems above, there are a second set of transport systems, generally dedicated to specific essential metals. In contrast to the general metal response system, which regulates metal ions during metal dyshomeostasis, these essential metal transport systems are active during stressful and non-stressful conditions to maintain homeostasis and transport the essential metals within the cell. These pathways are activated in tandem with the general metal response and, although they often have a primary role in a single metal, each pathway can interact with, and possibly regulate, other essential and non-essential metals, overlapping in their function [30,31,38]. Overall, metal specific transport systems effectively regulate the concentration of specific essential metal ions in the cell, while also indirectly aiding in maintaining the concentration of other essential and non-essential metal ions.

### 6.1. Copper Transport in Drosophila melanogaster

**Figure 2:**
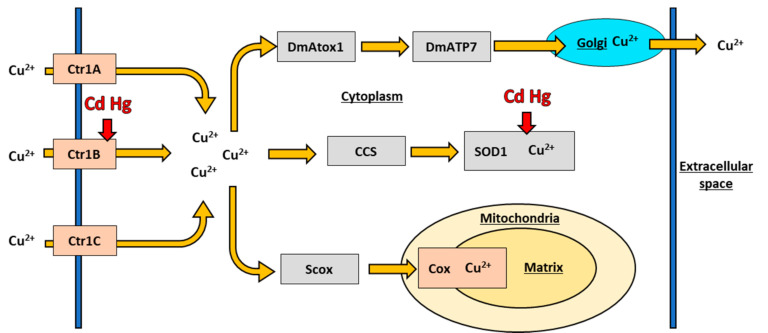
A diagram of the *D. melanogaster* Cu transport system transporting copper and interactions with other metals. In addition to the pattern and colour scheme from Figure 1, membrane proteins are tan, the mitochondria is pale yellow, the matrix is yellow, the Golgi apparatus is light blue, and metals, other than copper, that interact with proteins in the system are in red. Underlined text denote areas inside the cell, organelles, or space outside of the cell.

Copper is an essential metal which functions as a cofactor for superoxide dismutase (SOD) and cytochrome c oxidase (COX) in *D. melanogaster*, and other organisms [30,89]. The high redox potential of Cu facilitates the redox reactions catalyzed by these proteins in removing reactive oxygen species from the cell and transporting electrons in the electron transport chain [90]. The high potential also results in pronounced toxicity if Cu is unbound in the cytosol [31]. Organisms have a dedicated Cu transport system to regulate and transport Cu and balance the trade-off between function and toxicity which is conserved across distantly related species [30,38]. The *D. melanogaster* Cu transport system is shown below in Figure 2.

*D. melanogaster* have a family of Cu importer proteins. Cu is first imported into the cell by the Cu importers Ctr1A, Ctr1B, and Ctr1C [59]. The *D. melanogaster* Ctr1 family of proteins all regulate Cu import into the cell and are homologous to the human Ctr1 protein with which they have a conserved function and structure [59]. This conservation is sufficiently pronounced that expression of the human *Ctr1* gene will rescue knock out of the *D. melanogaster* *Ctr1A* gene [91], emphasizing the protein’s fundamental importance in Cu biology. The key difference between human *Ctr1* and the *D. melanogaster* *Ctr1* genes lies in the sub functionalization of the three paralogous *D. melanogaster* *Ctr1* genes.

Each of the three proteins import Cu into the cell, but each is specialized to have differential expression throughout the life cycle of the fly, differential expression to different tissue types, and different expression to metal stress [59,92,93]. Ctr1A, necessary to fly development and survival, is the primary isoform maintaining basal Cu import throughout the life of the fly [59]. Knockout of *Ctr1A* prevents larval development into adulthood, even on Cu supplemented food, and expression of *Ctr1A* transgenes rescues survival [92], indicating that Ctr1A is vital for basal fly development [92]. In contrast, Ctr1B is mainly responsible for controlling dietary Cu uptake in situations of metal stress [56,59,70]. *Ctr1B* is inhibited significantly more than *Ctr1A* and *Ctr1C* in situations of Cu toxicity [52,61] and upregulated significantly more than *Ctr1A* and *Ctr1C* during Cu starvation [56]. Interestingly, expression of the *D. melanogaster Ctr1C* gene is sexually dimorphic. *Ctr1A* and *Ctr1B* are expressed similarly in males and in females, but only *Ctr1C* is expressed in maturing spermatozoa and mature sperm [94]. Ctr1C is not, however, required for male fly fertility, and male flies with knockout mutations in *Ctr1C* or *Ctr1B*, independently, do not show a decrease in fertility, although double knockout flies are sterile [94]. Overall, the three Cu transport paralogs in *D. melanogaster* each play a distinct yet overlapping role regulating Cu import in the fly and are essential to understanding Cu and metal regulation in *D. melanogaster*.

### 6.2. Copper Chaperones in Drosophila melanogaster

Once Cu is imported into the cell by the Ctr family, intracellular Cu chaperones shuttle the ions to the appropriate proteins to act as co-factors, or export Cu ions from the cell if they are not required. DmAtox1 transports Cu to DmATP7 which then transports the Cu ions to the appropriate organelle for distribution or excretion [57,95,96]. *DmAtox1* is the *D. melanogaster* homolog of the human *Atox1* gene, while *DmATP7* is the *D. melanogaster* homolog to the human *ATP7A* and *ATP7B* genes [61,95]. When DmATP7 was introduced into mammalian cells*,* DmATP7 was able to translocate to the plasma membrane and partially restore function, suggesting a conservation of function between mammalian *ATP7A* and *B* and *D. melanogaster DmATP7*. Disruption to either *DmATP7* or *DmAtox1* leads to accumulation of Cu in the fly cytoplasm and Cu toxicity, suggesting that DmATP7 and DmAtox1 is the main route of efflux in *D. melanogaster* [57,95]. Intracellular Cu ions can also be transported by copper chaperone (CCS) to SOD1 along a pathway conserved from flies to mammals. Cu can then act as a metal cofactor for SOD1 [97,98]. Transfections of human *CCS* and human *Ctr1* have rescued fly survival on toxic Cu conditions [97], suggesting that either protein can support SOD1 function, allowing the fly to combat the resulting oxidative stress from the Cu toxic conditions. Finally, cytosolic Cu can be transported by Scox, a *D. melanogaster* ortholog to the mammalian protein SCO, which transports Cu to the mitochondrial protein COX [96]. COX is vital to the electron transport chain and Scox is present throughout the development of the fly. Unsurprisingly, when *Scox* is inhibited in *D. melanogaster*, flies do not develop to adulthood [99]. Proper Cu homeostasis is a function of all of these proteins working together, and knockout of any one portion of the Cu transport system significantly impacts fly survival and development.

### 6.3. Zinc Transport in Drosophila melanogaster

Zn is a redox-neutral essential metal [100] with a central role in determining protein structure; approximately 10% of the *D. melanogaster* proteome is Zn binding, and this percentage is consistent across other organisms [101]. Unlike Cu and Fe, which are both located in catalytic centres in their respective metalloproteins, Zn primarily functions to maintain a specific 3D structure in a protein by coordinating with cysteine and histidine residues in a zinc-finger sequence [102]. Zn metalloproteins act in a wide variety of different processes including reaction catalysis, immune function, cell signaling, and DNA synthesis [103]. Interestingly, high Zn concentrations can lead to Cu deficiency and Fe deficiency [71,72]**,** underscoring the interconnectedness of essential metal homeostasis in biology. Although cytosolic Zn ions are not redox active in the same way as Cu and Fe, Zn’s broad importance in protein structure requires strict maintenance through its own transport system.

**Figure 3:**
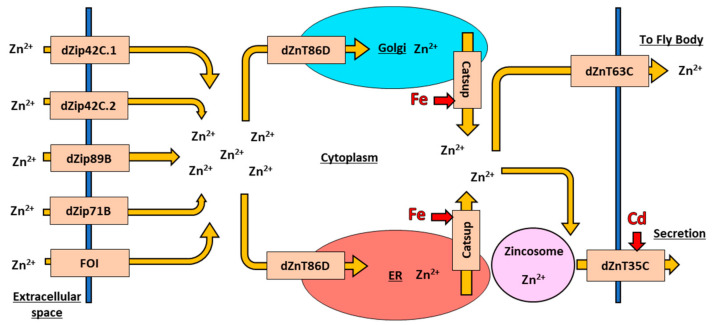
A diagram of the *D. melanogaster* Zn transport system transporting Zn and interactions with other metals. In addition to the pattern and colour schemes from Figure 1 and Figure 2, the endoplasmic reticulum is coloured blood orange, and extracellular transporters are coloured pink. Underlined labels denote areas inside the cell, organelles, or space outside of the cell.

In *D. melanogaster*, Zn homeostasis is maintained by eliminating excess cellular Zn to the Malpighian tubules, and reabsorbing Zn back into the body from the tubules as required [104], similar to mammalian kidney function. At the molecular level, *D. melanogaster* Zn homeostasis is maintained by two major protein families: ten Zn importer proteins (dZip) and seven Zn exporter proteins (dZnT) [105], working together to balance intracellular concentrations. The Zn transport system is well conserved between *D. melanogaster* and humans, with many dZip and dZnT having functional orthologs in the 24 human Zn importer proteins (ZIP) and Zn exporter proteins (ZnT) proteins [106]. The variety of currently studied importers and exporters in *D. melanogaster* is shown below in Figure 3.

### 6.4. Zinc Import in Drosophila melanogaster

The *D. melanogaster* dZip Zn importer protein family includes two different roles in the cell: import of extracellular Zn across the plasma membrane into the cytoplasm and import of intracellular Zn from organelles into the cytoplasm [30]. The first group of extracellular Zn importers includes three genes that are primarily expressed in the midgut of *D. melanogaster* to intake dietary Zn: *dZip42C.1*, *dZip42C.2*, and *dZip89B* [107,108]. The three proteins have some amount of functional overlap and knockout of any one gene results in overexpression of the other two without impact fly survival or health [107]. dZip71B, another extracellular Zn importer, shuttles Zn into the Malpighian tubules for export from the fly, and tubules-specific knockout of *dZip71B* causes Zn sensitivity and toxicity in the fly [104]. An extracellular Zn importer, the Fear-of-Intimacy (FOI) protein, has a specific role in morphogenesis of the trachea and gonads [109,110,111]. Elimination of *FOI* function results in the failure of gonad and tracheal tissues to develop properly during the larval phase, even though the cells themselves still differentiate and develop in the larvae [111]. The specific role of FOI is unknown, but the protein interacts with other downstream developmental proteins necessary for cell morphogenesis [111], so it is likely that FOI is required for proper extracellular Zn intake into *D. melanogaster* to drive proper fly development [109,112].

The second group of Zn importers are intracellular Zn importers, which are important for maintaining proper protein trafficking within the cell. The intracellular Zn importer protein Catsup is the *D. melanogaster* homolog to mammalian ZIP7, and drives the intake of Zn into the cytoplasm to mediate protein trafficking and intercellular cell signaling [38,113]. Disruption of the *Catsup* gene in *D. melanogaster* leads to an accumulation of Zn in the endoplasmic reticulum (ER) and Golgi apparatus, and a decrease in protein signaling by accumulating Notch in the ER, which affects downstream cell specification [114]. Interestingly, Catsup function also impact Fe homeostasis; elimination of Catsup activity results in an increase in Fe-dependent enzymatic activities in the cytosol [38], underscoring the interconnectedness of these metals although it is unclear if Catsup interacts directly or indirectly with Fe.

In a interesting twist, *D. melanogaster dZip13*, the homolog of human *ZIP13*, was initially classified as an intracellular Zn importer, but experiments indicate the primary function of the protein is as an Fe transporter [115,116]. The dZip13 protein does interact with both Fe and Zn, and interestingly dZip13 binds more readily to Zn in vitro [115], which is unexpected given its primary role as an Fe importer. Although dZip13 functions primarily as an Fe importer, its Zn binding capabilities and initial classification as a Zn importer leave room for the possibility that both metals interact with dZip13 in the regulation of Fe and/or Zn.

### 6.5. Zinc Export in Drosophila melanogaster

Similar to the Zn importers, Zn exporters can be separated into intracellular and extracellular proteins [30]. There are three particularly well studied *D. melanogaster* extracellular Zn exporters: dZnT63C, dZnT35C, and dZnT86D. Present in the plasma membrane of gut cells and Malpighian cells, dZnT63C plays a central role in maintaining Zn distribution across the body of the fly by exporting dietary Zn to the body of the fly and exporting Zn from the Malpighian tubules for reabsorption [106,117]. Suppression of *dZnT63C* results in accumulation of Zn in the gut lumen and concurrent Zn deficiency across the rest of the fly body, reflecting the broad role of dZnT63C in maintaining body-wide Zn homeostasis [108]. dZnT35C is also present in the Malpighian tubules, where it functions to create Zn storage granules crucial to Zn secretion and elimination from the cell [118,119]. Knockout of *dZnT35C* causes Zn sensitivity and accumulation in the fly, suggesting that Zn is eliminated through Zn storage granules and not ionic form that would be processed by dZnT63C. dZnT86D is present in the ER and Golgi apparatus and, given the role of export into these two organelles, it is often studied in combination with the Zn importer Catsup. Paradoxically, overexpression of *dZnT86D* produces toxic phenotypes normally associated with elevated, not lowered, cytosolic Zn levels, though this is likely as the toxic phenotypes are driven by elevated zinc concentrations in the Er and Golgi [106,113]. dZnT86D may also be involved in Fe regulation, given that dZnT86D is closely associated with Catsup, but current studies suggest that dZnT86D has no effect on the Fe regulatory properties of Catsup [120].

The Zn transport system in *D. melanogaster* maintains Zn homeostasis using a suite of conserved Zn importers and exporters, each performing specific functions within the cell and the fly as a whole. Further research is needed to explore other less studied importers and exporters such as dZip48C, dZip102B, dZnT33D, dZnT77C, and dZnT41, and the interactions with other essential and non-essential metals.

### 6.6. Iron Transport in Drosophila melanogaster

**Figure 4:**
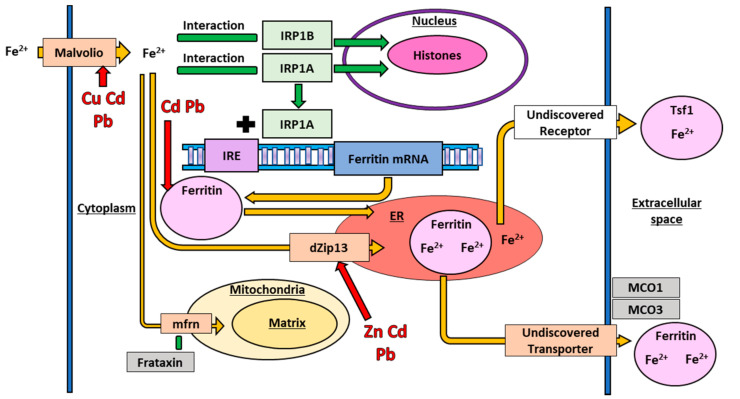
A diagram of the *D. melanogaster* Fe transport system transporting Fe and interactions with other metals. In addition to the pattern and colour scheme of all previous figures, histones are coloured magenta in our diagram, mRNA is coloured blue, and receptors are coloured white. Underlined labels denote areas inside the cell, organelles, or space outside of the cell.

Fe is an essential metal that functions as a redox center in many metalloproteins which are required for a variety of cellular processes including cell signaling, movement of electrons in the electron transport chain (ETC), immune function, and DNA synthesis [88,121]. As with Cu, Fe has a high redox potential, and ferrous Fe is very toxic to the cell. The toxicity is primarily through the production of ROS in the cytoplasm through the Fenton reaction, and organisms have evolved a series of systems to balance the tradeoff between the essential functions of Fe and its toxicity. The transport of Fe in *D. melanogaster* is shown below in Figure 4.

### 6.7. Iron Import in Drosophila melanogaster

In *D. melanogaster*, the Malvolio protein imports extracellular Fe into the cell cytoplasm. *Malvolio* is the insect homolog to the mammalian Fe import gene *Divalent metal transporter-1* (*DMT-1)* [121]; the protein is well conserved across distantly related taxa [122,123]. *Malvolio* is highly expressed in the midgut where it functions in dietary Fe absorption and the Malpighian tubules where it functions in Fe excretion [123]. Elimination of *Malvolio* reduces total Fe in the fly, but only by approximately 25% [121], suggesting that *D. melanogaster* has other Fe importer proteins or means of importing Fe, although none have been identified to date. Malvolio is also capable of importing other divalent metal ions, including Cu [124] and the protein appears to work with the Ctr1 family of proteins to maintain proper Cu concentrations [124].

Once Fe is in the cytoplasm, it interacts with a suite of regulatory proteins which regulate the transcription and translation of various metal response genes. In mammalian cells, Fe interacts with iron regulatory protein-1 (IRP1) and iron response elements (IRE) to stabilize the mRNA of associated Fe response genes and allow mRNA translation in response to changing Fe conditions in the cell [125]. In *D. melanogaster*, Fe interacts with dMTF-1, which we have discussed earlier, and the Fe-specific iron regulatory protein-1A (IRP1A) and B (IRP1B) [126]. *IRP1A* and *IRP1B* are paralogs, and sequence analysis of the two *D. melanogaster* homologues demonstrate a striking sequence similarity to vertebrate *IRP1* [126]. Interestingly, *D. melanogaster* IRP1A can bind both human and *D. melanogaster* IRE, whereas IRP1B is unable to bind to either IRE, suggesting greater conservation of function between the mammalian protein and IRP1A than IRP1B [127]. IRP1A then binds to an IRE situated upstream of a ferritin mRNA to regulate translation [127]. Recent work has shown that IRP1A and IRP1B both also translocate to the nucleus where they bind to histones to regulate proteins involved in Fe homeostasis [128]. Further research into the interactions across all these proteins will improve the understanding of Fe homeostasis in *D. melanogaster*.

### 6.8. Iron Involvement in Drosophila melanogaster Spermatogenesis

Fe metabolism also has a sex-specific component; the transport protein mitoferrin (mfrn) is required for proper Fe transport into the *D. melanogaster* mitochondria during spermatogenesis [129]. The mechanism of transport into the mitochondria, and any additional functions for mfrn are currently unknown, but may involve frataxin, another putative Fe response protein. Frataxin appears to be involved in ROS response [130] and frataxin deficient flies are a model for mitochondrial Fe accumulation [131]. Interestingly, downregulation of *mfrn* in these flies ameliorated disease phenotypes [132]. Identification of the mechanism of this interaction will require additional research.

### 6.9. Iron Storage and Export in Drosophila melanogaster

Multicopper oxidases (MCO) are ferroxidases that oxidize redox-active ferrous Fe to ferric Fe which is needed in other downstream processes [133,134]. MCO’s are vital Fe exporters in mammals [135,136], but the *D. melanogaster* homologs primarily function in Fe storage, prevention of Fe toxicity, and ROS production [121,135,136,137]. *D. melanogaster* has four paralogous *MCO* genes (*MCO1–4*). MCO1 has a major role in maintaining Fe homeostasis; however, the mechanism through which the protein maintains homeostasis is not through ferroxidation, but instead through a separate, and currently unknown, indirect mechanism [135,136]. MCO3 also impacts Fe homeostasis, as *MCO3* mutant flies accumulate Fe in the cytosol and experience decreased *Mvl* expression, but in contrast to MCO1, MCO3 has confirmed ferroxidase activity [121,135]. Current research into the involvement of MCOs in *D. melanogaster* metal homeostasis is promising, but more experimental data exploring the mechanisms and potentially yet to be discovered genes is needed.

In mammalian cells, ferric Fe is transported out of the cytoplasm by an exporter in the plasma membrane called ferroportin [138], but no homologs for ferroportin have been discovered at this time in *D. melanogaster* [139]. Fe is likely transported out of the cytosol and into the ER via dZip13, where it binds to ferritin, and ferritin is then transported out of the cell [115,139]. Ferritin, an Fe storage protein, is the most well studied of these three proteins [137,140]. In *D. melanogaster*, ferritin functions in both an Fe storage and transport of dietary Fe from gut cells to the rest of the fly body [140,141]. Without ferritin present, dietary Fe is not distributed to the rest of the fly, and Fe is not secreted from cells when it reaches toxic concentrations [139,140]. Additionally, ferritin is vital to the *D. melanogaster* immune response, as ferritin sequesters Fe, which is needed for pathogen proliferation [121]. Transferrin-1 (Tsf1) is another possible exporter of Fe and the *D. melanogaster* homolog to mammalian *transferrin* (*TF*) which binds ferric Fe in mammals for transport by binding to cell surface receptors TF receptor 1 and 2 [139]. Similar to ferritin, Tsf1 transports Fe in *D. melanogaster* from the gut to the body, but the Fe binding mechanism is unknown, given that no *D. melanogaster* TF receptors have been discovered [139]. As discussed above, dZip13 is stabilized by Fe and is involved in Fe homeostasis via ferritin formation [115]. The functional similarity between ferritin and Tsf1 in Fe transport suggests a possible interaction between Tsf1 and dZip13, but the interaction remains to be demonstrated [139]. Overall, while ferritin, Tsf1, and dZip13 are all involved in Fe export, more research is needed to elucidate their how they, and potentially additional proteins, interact to effectively regulate Fe homeostasis.

## 7. Non-Essential Metals Interacting with Essential Metal Pathways

We have presented the above systems as a series of pathways regulating individual metals, but the functional reality likely involves interactions and interconnections across pathways, including between essential and non-essential metal pathways. For example, transcription of the *D. melanogaster* Cu importer gene *Ctr1B*, is downregulated in response to Cu toxicity and upregulated in situations of Cu starvation, but the gene is also upregulated in response to toxicity from non-essential metals such as Cd and Hg [64]. The upregulation seems paradoxical in that it could also lead to further ROS production from Cu ions, in addition to that produced by the non-essential metals. However, increased Ctr1B activity and increased Cu concentration leads to increased production of SOD1, which transforms the superoxide radical produced by the non-essential metals to the comparatively less toxic hydrogen peroxide [64]. Activity across these pathways appears to be a delicate balancing around total ROS.

The Zn and Fe transport systems also interact with non-essential metals. The extracellular Zn exporter *dZnT35C* is upregulated in response to Cd toxicity, possibly exporting Cd ions via Zn storage granules [118]. The system may function to limit non-essential metal toxicity by using storage molecules such as Zn storage granules. Similarly, ferritin, an Fe storage protein, is also upregulated in response to non-essential metal toxicity [142,143]. For example, Cd and Pb toxicity both increase dZip13 activity, which increases ferritin production [143]. Ferritin production is typically linked to transport and storage of Fe. Pb and Cd toxicity impairs Fe transport in *D. melanogaster* cells suggesting a connection [143]. Pb and Cd toxicity also upregulates expression of *Mvl*, another extracellular Fe importer [143] suggesting a complicated set of interactions with Fe metabolism proteins. The increased ferritin may reduce oxidative stress created by non-essential metals [142], or may be able to bind to non-essential metals [144] for secretion from the cell as well, but it is also possible that the increased ferritin production is the result of an unintended crosstalk or breakdown of the system under toxic conditions. The interaction across these pathways underscores the complexity of the biology of essential and non-essential metal homeostasis in *D. melanogaster* and the need for broader research investigating multiple essential metal pathways, and the use of non-essential metals research in essential metal pathways. A summary of proteins discussed in this paper which interact with multiple metal ions is presented below in Table 1.

## 8. Conclusions and Future Directions

In biology, metal ions are both necessities and toxicants. While an excellent foundation of research exists, there are still many unanswered questions. Given the central role of many metal ions and the global issues of environmental metal contamination, future research to address these questions is crucial. *D. melanogaster* is an excellent experimental model organism for the study of the effects of metal toxicity, and the broad evolutionary conservation in systems that have been explored suggest that studies of this species will shed light on the fundamental biology of metal toxicity for both essential and non-essential metals. Future research is particularly needed in identifying still missing pieces of these pathways, for example a *D. melanogaster* homolog to the human TF receptor, and in determining the mechanisms of toxicity and interactions with both the general and metal specific response systems.

This review highlights the current knowledge of essential metals (Fe, Zn, and Cu) and various non-essential metals (Ni, Hg, Pb, and Cd) in *D. melanogaster*. However, there are other lesser studied essential metals in *D. melanogaster* including manganese, cobalt, and molybdenum which are not covered in this review. Each of these three essential metals interact with other essential metals and pathways [145,146,147]. There are other areas of biology to which metals are vital that were not discussed in this review, such as immune response, DNA synthesis, etc. Elucidating the mechanisms in the metal pathways will improve the understanding of broader metal biology.

Metal response is a complex web. Metals do not exist in isolation, and dyshomeostasis in one affects not only its own pathway, but other essential and non-essential metal pathways as well, and the general metal response proteins [38,80]. Given these interactions, it is crucial that future toxicity research examines combinations of essential and non-essential metals. Some studies have already compared multiple metal ions in the same experiment such as Cu, Zn, Fe, and Cd [35,48,54,148] to great success. Studies including both essential and non-essential metals will allow comparison of chemically similar, but biologically distinct, metal ions. Finally, the effects of sex and genetic background on metal toxicity are understudied. Research in *D. melanogaster* clearly shows significant and substantial variation between the sexes and across genetic backgrounds, and it is crucial that future studies explore these if we are to understand the true biology of metal toxicity.

## Figures and Tables

**Table 1:** *D. melanogaster* metal transport systems interacting with multiple essential and non-essential metals.

System	Gene	Interactions with Metal Ions	References
General Metal Response System	*dMTF-1*	Cu, Zn, Fe, and Cd	[35,42,48,50,51,58,59,60]
	*MtnA-F*	Cu, Zn, Fe, Cd, Pb, and Hg	[35,40,50,52,54,64,69,70,78,81,84]
Copper Transport System	*Ctr1B*	Cu, Cd, and Hg	[64]
	*SOD1*		
Zinc Transport System	*Catsup*	Zn and Fe	[38]
	*dZnt35C*	Zn and Cd	[118]
Iron Transport System	*Malvolio*	Fe, Cu, Cd, and Pb	[124,143]
	*Ferritin*	Fe, Cd, and Pb	[142,143,144]
	*dZip13*	Fe, Zn, Cd, and Pb	[115,143]

## Data Availability

Not applicable.
